# Serial diffusion tensor MRI and tractography of the mouse heart in-vivo: impact of ischemia on myocardial microstructure

**DOI:** 10.1186/1532-429X-13-S1-O28

**Published:** 2011-02-02

**Authors:** Shuning Huang, Choukri Mekkaoui, Howard H Chen, Roupeng Wang, Soeun Ngoy, Ronglih Liao, Van Wedeen, Guangping Dai, David E Sosnovik

**Affiliations:** 1 Martinos Center for Biomedical Imaging, Massachusetts General Hospital, Charlestown, MA, USA; 2 Cardiology Division, Brigham and Woman’s Hospital, Boston, MA, USA

## Objective

To perform serial in-vivo diffusion tensor MRI (DTI) and tractography of the mouse heart and characterize the impact of ischemia on myocardial microstructure.

## Background

Diffusion tensor MRI (DTI) has been used to investigate infarct healing and remodeling in several species. Tractography of the myocardium has also recently been reported. However, these prior studies were conducted ex-vivo on excised hearts. Here, we use in-vivo DTI of the mouse heart to follow microstructural changes in the myocardium in response to ischemia. Mean diffusivity (MD) and fractional anisotropy (FA) were measured 24 hours and 2-3 weeks after ischemia-reperfusion. In addition, 3D diffusion MRI tractography of the mice hearts was performed *in-vivo*.

## Material and methods

11 mice were used: Six as normal controls and five exposed to 35min left coronary artery ligation followed by reperfusion. In vivo DTI was performed 24 hours and 2-3 weeks post-injury on a 9.4T scanner (Biospin, Bruker) with a 150 Gauss/cm gradient. The in- vivo DTI sequence was based on a fat-suppressed single-shot spin echo EPI sequence with motion-compensated bipolar diffusion-encoding gradients on either side of the 180° RF pulse. Imaging parameters included: FOV: 2.0 x 2.0 cm^2^, matrix: 70 x 70 (padded to 128 x 128), TR/TE: 2000/13.5 ms, b-value 500 - 700 sec/mm^2^. 3D parameters were similar but with an isotropic resolution of 280 um.

## Results

**Figure 1:**
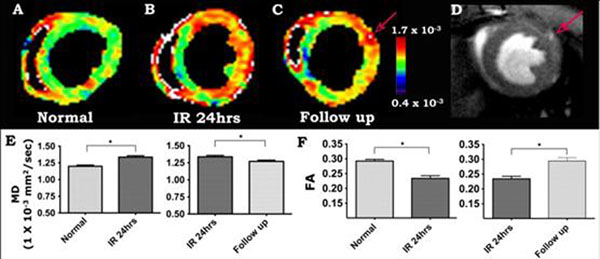
Serial in-vivo DTI of the mouse heart. Each mouse was imaged in 24hrs and 2-3wks after ischemia reperfusion. The mean diffusivity (MD) maps clearly showed that MD in the injured anternolateral wall increased acutely (24hrs after injury) and subsequently returned towards baseline as the myocardium healed (A, B, C, E). Corresponding to the changes in MD, fractional anisotropy (FA) decreased acutely and returned to baseline 2-3wks after injury (F). (C-D) The left coronary artery, when present in the image, can be used for internal standardization and control. MD in the vessel and its branches (red arrow, C, D) will be significantly higher than MD in normal or healing myocardium.

**Figure 2:**
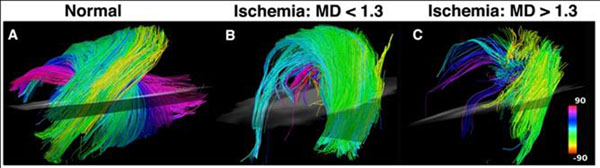
In-Vivo diffusion MRI tractography of mouse hearts. Myofibers passing through a region-of interest placed in the antero-lateral wall are shown. The fibers are color-coded by the helix angle they make with the left ventricle. (A) Normal mouse heart. The characteristic arrangement of myofibers into an array of crossing helices is clearly seen. (B) Mouse exposed to ischemia-reperfusion with severe hypokinesis in it anter-lateral wall. Mean diffusivity (MD), however, has remained < 1.3 x 10^-3^ mm^2^/sec and fiber architecture remains reasonably organized. (C) Mouse exposed to ischemia-reperfusion, also presenting with severe hypokinesis of the anterolateral wall. MD in this mouse, however, is < 1.3 x 10^-3^ mm^2^/sec and fiber architecture is severely perturbed.

A significant (* p < 0.05) increase in MD and decrease in FA were seen in mice 24 hours after ischemia-reperfusion (Figure 1). Significant differences in MD and FA were also seen between the ischemic mice at 24hrs and 2-3 weeks of follow-up (Figure 1). In-vivo tractography produced tractograms of similar quality to those previously acquired ex-vivo, with the characteristic crossing myofiber helices well seen in normal mice (Figure 2A). Myofiber architecture was markedly perturbed in the ischemic mice (Figure 2B-C). A large increase in MD (>1.3 x 10^-3^ mm^2^/sec) at 24 hours correlated strongly with a severe loss of myofiber architecture (Figure 2B-C).

## Conclusion

Combined in-vivo DTI and tractography of the myocardium is presented for the first time. The technique shows that MD increases and FA decreases in acute ischemia, and thereafter return towards baseline as the myocardium heals. The loss of myofiber architecture in acute ischemia is variable, but is marked when MD is significantly elevated. Ongoing use of these techniques in-vivo has the potential to become an extremely powerful tool in cardiovascular MRI.

